# Supplementary material for: Ten months of temporal variation in the clinical journey of hospitalised patients with COVID-19: An observational cohort
Source: eLife. 2021 Nov 23;10:e70970. doi: 10.7554/eLife.70970 (PMC8791638; doi:10.7554/eLife.70970)
Supplement: Supplementary file 1. [file elife-70970-supp1.docx]

Dependent variables used were:

- Month of COVID-19 admission, or, for question 1, month of symptom onset, as a discrete variable. April was used as the reference category; this is because the first month chronologically, March, was an outlier in many ways and comparisons with that month tended to give universally small *p*-values when compared to other months.
- Sex
- Final outcome (death or discharge)
- Age group (at admission), defined as 0-19, 20-39, 40-59, 60-69, 70-79, or 80+. The 40-59 age group was used as the reference category,
- For questions 2 to 5, either the time from symptom onset to hospital admission, or a variable indicating a nosocomial infection. This was binned as follows: nosocomial infection, 0-6 days, 7-13 days, and 14 days or greater. 0-6 days was the reference category.
- Country. In each analysis, countries with less than 200 patients represented were grouped by continent. Note that, due to differences in recruitment procedures between sites in the consortium, estimated regression coefficients for this variable should not be taken as representative of overall differences in clinical care between countries, nor of demographic differences.
- The four most common symptoms recorded at admission were cough, fatigue, fever, and shortness of breath. For question 1, the number of these that were reported at hospital admission was counted, giving a score from 0 to 4. Missing data on these was counted as absence. It was excluded from questions 2 to 5 because it proved to be highly collinear with time from onset to admission (see Results).
- Recorded pregnancy in female patients; this was coded as an interaction term with sex such that comparisons were made only with non-pregnant female patients.
- A wide variety of comorbidities were also recorded (see table S1). As these data were frequently not recorded, such that treating unknown values as missing would severely decrease the size of the dataset used in the regression analyses, we allowed these variables to take separate values of “Present”, “Absent” and “Unknown”. These were not used in analyses 1 or 6.
