# Supplementary material for: Ten months of temporal variation in the clinical journey of hospitalised patients with COVID-19: An observational cohort
Source: eLife. 2021 Nov 23;10:e70970. doi: 10.7554/eLife.70970 (PMC8791638; doi:10.7554/eLife.70970)
Supplement: Supplementary file 8. [file elife-70970-supp8.docx]

**SUPPLEMENTARY MATERIAL - ISARIC Clinical Characterisation Group***

**AUTHORS**

**CRediT author statement (based on Brand et al., 2015, doi: 10.1087/20150211)**

**Conceptualization** (this analysis): Matthew Hall, Gail Carson, Piero L. Olliaro, Amanda Rojek.

**Methodology**: Matthew Hall, Christl A. Donnelly, Christiana Kartsonaki.

**Software and formal analysis**: Matthew Hall; Emmanuelle Dankwa, Christiana Kartsonaki, Kenneth A. McLean, Mark Pritchard.

**Conceptualization:** (study) J. Kenneth Baillie, Husna Begum, Aidan Burrell, Gail Carson, J Perren Cobb, Jake Dunning, Robert A. Fowler, Peter Horby, John Marshall, Colin McArthur, Laura Merson, Srinivas Murthy, Alistair Nichol, Rachael Parke, Malcolm G. Semple, Timothy M. Uyeki, Steve Webb.

**Data curation**: Barbara Wanjiru Citarella, Sadie Kelly, Kalynn Kennon, James Lee, Laura Merson, Daniel Plotkin, Sue Smith, Samantha Strudwick.

**Administration**: Barbara Wanjiru Citarella, Laura Merson.

**Writing - original draft**: Matthew Hall, Joaquín Baruch, Andrew Dagens, Jake Dunning, Christiana Kartsonaki, Piero L. Olliaro, Amanda Rojek.

**Visualization**: Matthew Hall.

**Writing - review and editing:** All authors.

**Data Contributors- including roles of Investigation, Data curation, Supervision, Resources, Project administration, Methodology, Formal analysis and Funding acquisition, Software, Validation, Visualization, and Conceptualization:** Sheryl Ann Abdukahil, Ryuzo Abe, Laurent Abel, Lara Absil, Subhash Acharya, Andrew Acker, Shingo Adachi, Elisabeth Adam, Diana Adrião, Saleh Al Ageel, Shakeel Ahmed, Kate Ainscough, Ali Ait Hssain, Younes Ait Tamlihat, Takako Akimoto, Ernita Akmal, Eman Al Qasim, Razi Alalqam, Tala Al-dabbous, Senthilkumar Alegesan, Cynthia Alegre, Beatrice Alex, Kévin Alexandre, Abdulrahman Al-Fares, Huda Alfoudri, Imran Ali, Kazali Enagnon Alidjnou, Jeffrey Aliudin, Qabas Alkhafajee, Clotilde Allavena, Nathalie Allou, João Alves, João Melo Alves, Rita Alves, Maria Amaral, Heidi Ammerlaan, Phoebe Ampaw, Roberto Andini, Claire Andrejak, Andrea Angheben, François Angoulvant, Séverine Ansart, Massimo Antonelli, Carlos Alexandre Antunes de Brito, Ardiyan Apriyana, Yaseen Arabi, Irene Aragao, Francisco Arancibia, Carolline Araujo, Antonio Arcadipane, Patrick Archambault, Lukas Arenz, Jean-Benoît Arlet, Christel Arnold-Day, Lovkesh Arora, Rakesh Arora, Elise Artaud-Macari, Diptesh Aryal, Motohiro Asaki, Angel Asensio, Sheharyar Ashraf, Jean Baptiste Assie, Anika Atique, AM Udara Lakshan Attanyake, Johann Auchabie, Hugues Aumaitre, Adrien Auvet, Laurène Azemar, Cecile Azoulay, Benjamin Bach, Delphine Bachelet, Claudine Badr, Nadia Baig, J. Kenneth Baillie, Erica Bak, Agamemnon Bakakos, Andriy Bal, Valeria Balan, Firouzé Bani-Sadr, Renata Barbalho, Wendy S. Barclay, Michaela Barnikel, Helena Barrasa, Audrey Barrelet, Cleide Barrigoto, Marie Bartoli, Cheryl Bartone, Joaquín Baruch, Romain Basmaci, Denise Battaglini, Jules Bauer, Diego Fernando Bautista Rincon, Abigail Beane, Alexandra Bedossa, Sylvie Behilill, Aleksandr Beljantsev, David Bellemare, Anna Beltrame, Marine Beluze, Nicolas Benech, Dehbia Benkerrou, Suzanne Bennett, Luís Bento, Jan-Erik Berdal, Delphine Bergeaud, Hazel Bergin, José Luis Bernal Sobrino, Giulia Bertoli, Lorenzo Bertolino, Simon Bessis, Adam Betz, Sybille Bevilcaqua, Karine Bezulier, Amar Bhatt, Krishna Bhavsar, Isabella Bianchi, Claudia Bianco, Moirangthem Bikram Singh, Felwa Bin Humaid, François Bissuel, Patrick Biston, Laurent Bitker, Pablo Blanco-Schweizer, Catherine Blier, Frank Bloos, Mathieu Blot, Filomena Boccia, Laetitia Bodenes, Alice Bogaarts, Debby Bogaert, Anne-Hélène Boivin, Pierre-Adrien Bolze, François Bompart, Diogo Borges, Raphaël Borie, Hans Martin Bosse, Elisabeth Botelho-Nevers, Lila Bouadma, Olivier Bouchaud, Sabelline Bouchez, Dounia Bouhmani, Damien Bouhour, Kévin Bouiller, Laurence Bouillet, Camile Bouisse, Anne-Sophie Boureau, John Bourke, Maude Bouscambert, Aurore Bousquet, Jason Bouziotis, Bianca Boxma, Marielle Boyer-Besseyre, Maria Boylan, Matthew Brack, Axelle Braconnier, Cynthia Braga, Timo Brandenburger, Filipa Brás Monteiro, Luca Brazzi, Dorothy Breen, Patrick Breen, Patrick Breen, Kathy Brickell, Alex Browne, Shaunagh Browne, Nicolas Brozzi, Nina Buchtele, Christian Buesaquillo, Marielle Buisson, Erlina Burhan, Ingrid G. Bustos, André Cabie, Susana Cabral, Eder Caceres, Cyril Cadoz, Kate Calligy, Jose Andres Calvache, João Cam, Valentine Campana, Paul Campbell, Cecilia Canepa, Mireia Cantero, Pauline Caraux-Paz, Sheila Cárcel, Chiara Simona Cardellino, Filipa Cardoso, Filipe Cardoso, Nelson Cardoso, Sofia Cardoso, Simone Carelli, Nicolas Carlier, Thierry Carmoi, Gayle Carney, Chloe Carpenter, Inês Carqueja, Marie-Christine Carret, François Martin Carrier, Ida Carroll, Gail Carson, Ed Carton, Maire-Laure Casanova, Mariana Cascão, Siobhan Casey, José Casimiro, Bailey Cassandra, Silvia Castañeda, Nidyanara Castanheira, Guylaine Castor-Alexandre, Henry Castrillón, Ivo Castro, Ana Catarino, François-Xavier Catherine, Paolo Cattaneo, Roberta Cavalin, Giulio Giovanni Cavalli, Alexandros Cavayas, Adrian Ceccato, Minerva Cervantes-Gonzalez, Anissa Chair, Catherine Chakveatze, Adrienne Chan, Meera Chand, Christelle Chantalat Auger, Jean-Marc Chapplain, Julie Chas, Jonathan Samuel Chávez Iñiguez, Anjellica Chen, Yih-Sharng Chen, Matthew Pellan Cheng, Antoine Cheret, Thibault Chiarabini, Julian Chica, Catherine Chirouze, Davide Chiumello, Hwa Jin Cho, Sung-Min Cho, Bernard Cholley, Marie-Charlotte Chopin, Jose Pedro Cidade, Barbara Wanjiru Citarella, Anna Ciullo, Jennifer Clarke, Sara Clohisey, Necsoi Coca, Cassidy Codan, Caitriona Cody, Alexandra Coelho, Megan Coles, Gwenhaël Colin, Michael Collins, Sebastiano Maria Colombo, Pamela Combs, Jennifer Connolly, Marie Connor, Anne Conrad, Sofía Contreras, Elaine Conway, Graham S. Cooke, Mary Copland, Hugues Cordel, Amanda Corley, Sarah Cormican, Sabine Cornelis, Arianne Joy Corpuz, Grégory Corvaisier, Camille Couffignal, Sandrine Couffin-Cadiergues, Roxane Courtois, Stéphanie Cousse, Sabine Croonen, Gloria Crowl, Jonathan Crump, Claudina Cruz, Juan Luis Cruz Berm, Jaime Cruz Rojo, Marc Csete, Alberto Cucino, Ailbhe Cullen, Caroline Cullen, Matthew Cummings, Ger Curley, Elodie Curlier, Colleen Curran, Paula Custodio, Ana da Silva Filipe, Charlene Da Silveira, Al-Awwab Dabaliz, Andrew Dagens, Darren Dahly, Heidi Dalton, Jo Dalton, Seamus Daly, Federico D'Amico, Nick Daneman, Corinne Daniel, Emmanuelle A Dankwa, Jorge Dantas, Frédérick D'Aragon, Mark de Boer, Gillian de Loughry, Diego de Mendoza, Etienne De Montmollin, Rafael Freitas de Oliveira França, Ana Isabel de Pinho Oliveira, Rosanna De Rosa, Thushan de Silva, Peter de Vries, Jillian Deacon, David Dean, Alexa Debard, Bianca DeBenedictis, Marie-Pierre Debray, Nathalie DeCastro, William Dechert, Lauren Deconninck, Romain Decours, Eve Defous, Isabelle Delacroix, Eric Delaveuve, Karen Delavigne, Nathalie M. Delfos, Ionna Deligiannis, Andrea Dell'Amore, Christelle Delmas, Pierre Delobel, Elisa Demonchy, Emmanuelle Denis, Dominique Deplanque, Pieter Depuydt, Mehul Desai, Diane Descamps, Mathilde Desvallée, Santi Dewayanti, Alpha Diallo, Sylvain Diamantis, André Dias, Juan Jose Diaz, Priscila Diaz, Rodrigo Diaz, Kévin Didier, Jean-Luc Diehl, Wim Dieperink, Jérôme Dimet, Vincent Dinot, Fara Diop, Alphonsine Diouf, Yael Dishon, Félix Djossou, Annemarie B. Docherty, Helen Doherty, Arjen M Dondorp, Christl A. Donnelly, Maria Donnelly, Chloe Donohue, Sean Donohue, Yoann Donohue, Ciara Doran, Peter Doran, Céline Dorival, Eric D'Ortenzio, James Joshua Douglas, Renee Douma, Nathalie Dournon, Triona Downer, Joanne Downey, Mark Downing, Tom Drake, Aoife Driscoll, Claudio Duarte Fonseca, Vincent Dubee, François Dubos, Alexandre Ducancelle, Toni Duculan, Susanne Dudman, Abhijit Duggal, Paul Dunand, Jake Dunning, Mathilde Duplaix, Emanuele Durante-Mangoni, Lucian Durham III, Bertrand Dussol, Juliette Duthoit, Xavier Duval, Anne Margarita Dyrhol-Riise, Marco Echeverria-Villalobos, Siobhan Egan, Carla Eira, Mohammed El Sanharawi, Subbarao Elapavaluru, Brigitte Elharrar, Jacobien Ellerbroek, Philippine Eloy, Tarek Elshazly, Isabelle Enderle, Ilka Engelmann, Vincent Enouf, Olivier Epaulard, Martina Escher, Mariano Esperatti, Hélène Esperou, Marina Esposito-Farese, João Estevão, Manuel Etienne, Nadia Ettalhaoui, Anna Greti Everding, Mirjam Evers, Isabelle Fabre, Marc Fabre, Amna Faheem, Arabella Fahy, Cameron J. Fairfield, Pedro Faria, Nataly Farshait, Arie Zainul Fatoni, Karine Faure, Raphaël Favory, Mohamed Fayed, Niamh Feely, Laura Feeney, Jorge Fernandes, Marília Fernandes, Susana Fernandes, François-Xavier Ferrand, Eglantine Ferrand Devouge, Joana Ferrão, Mário Ferraz, Benigno Ferreira, Sílvia Ferreira, Ricard Ferrer-Roca, Nicolas Ferriere, Céline Ficko, Claudia Figueiredo-Mello, Juan Fiorda, Thomas Flament, Clara Flateau, Tom Fletcher, Letizia Lucia Florio, Brigid Flynn, Deirdre Flynn, Claire Foley, Jean Foley, Tatiana Fonseca, Patricia Fontela, Simon Forsyth, Denise Foster, Giuseppe Foti, Erwan Fourn, Robert A. Fowler, Dr Marianne Fraher, Diego Franch-Llasat, Christophe Fraser, John F Fraser, Marcela Vieira Freire, Ana Freitas Ribeiro, Caren Friedrich, Ricardo Fritz, Stéphanie Fry, Nora Fuentes, Masahiro Fukuda, Valérie Gaborieau, Rostane Gaci, Massimo Gagliardi, Jean-Charles Gagnard, Amandine Gagneux-Brunon, Sérgio Gaião, Linda Gail Skeie, Phil Gallagher, Elena Gallego Curto, Carrol Gamble, Arthur Garan, Rebekha Garcia, Noelia García Barrio, Esteban Garcia-Gallo, Denis Garot, Valérie Garrait, Nathalie Gault, Aisling Gavin, Anatoliy Gavrylov, Alexandre Gaymard, Johannes Gebauer, Eva Geraud, Louis Gerbaud Morlaes, Nuno Germano, Jade Ghosn, Marco Giani, Carlo Giaquinto, Jess Gibson, Tristan Gigante, Morgane Gilg, Elaine Gilroy, Guillermo Giordano, Michelle Girvan, Valérie Gissot, Judy Gitahi, Gezy Giwangkancana, Daniel Glikman, Eric Gnall, Geraldine Goco, François Goehringer, Siri Goepel, Jean-Christophe Goffard, Jonathan Golob, Rui Gomes, Joan Gómez-Junyent, Marie Gominet, Alicia Gonzalez, Patricia Gordon, Isabelle Gorenne, Laure Goubert, Cécile Goujard, Tiphaine Goulenok, Margarite Grable, Jeronimo Graf, Edward Wilson Grandin, Pascal Granier, Giacomo Grasselli, Lorenzo Grazioli, Christopher A. Green, William Greenhalf, Segolène Greffe, Domenico Luca Grieco, Matthew Griffee, Fiona Griffiths, Ioana Grigoras, Albert Groenendijk, Anja Grosse Lordemann, Heidi Gruner, Yusing Gu, Fabio Guarracino, Jérémie Guedj, Martin Guego, Dewi Guellec, Anne-Marie Guerguerian, Daniela Guerreiro, Romain Guery, Anne Guillaumot, Laurent Guilleminault, Maisa Guimarães de Castro, Thomas Guimard, Daniel Haber, Hannah Habraken, Ali Hachemi, Nadir Hadri, Olena Haidash, Sheeba Hakak, Adam Hall, Matthew Hall, Sophie Halpin, Ansley Hamer, Rebecca Hamidfar, Terese Hammond, Rashan Haniffa, Hayley Hardwick, Ewen M. Harrison, Janet Harrison, Samuel Bernard Ekow Harrison, Alan Hartman, Muhammad Hayat, Ailbhe Hayes, Leanne Hays, Jan Heerman, Lars Heggelund, Ross Hendry, Martina Hennessy, Aquiles Henriquez-Trujillo, Maxime Hentzien, Jaime Hernandez-Montfort, Daniel Herr, Andrew Hershey, Liv Hesstvedt, Astarini Hidayah, Dawn Higgins, Eibhilin Higgins, Grainne HigginsOKeeffe, Rita Hinchion, Samuel Hinton, Hiroaki Hiraiwa, Hikombo Hitoto, Antonia Ho, Alexandre Hoctin, Isabelle Hoffmann, Oscar Hoiting, Rebecca Holt, Jan Cato Holter, Peter Horby, Juan Pablo Horcajada, Koji Hoshino, Kota Hoshino, Ikram Houas, Catherine L. Hough, Stuart Houltham, Jimmy Ming-Yang Hsu, Jean-Sébastien Hulot, Samreen Ijaz, Hajnal-Gabriela Illes, Patrick Imbert, Hugo Inácio, Elias Iosifidis, Sarah Isgett, Palliya Guruge Pramodya Ishani Ishani, Tiago Isidoro, Margaux Isnard, Junji Itai, Daniel Ivulich, Danielle Jaafar, Salma Jaafoura, Julien Jabot, Clare Jackson, Nina Jamieson, Pierre Jaquet, Coline Jaud-Fischer, Stéphane Jaureguiberry, Issrah Jawad, Florence Jego, Synne Jenum, Ruth Jimbo-Sotomayor, Ruth N. Jorge García, Cédric Joseph, Mark Joseph, Swosti Joshi, Mercé Jourdain, Philippe Jouvet, Jennifer June, Anna Jung, Hanna Jung, Dafsah Juzar, Ouifiya Kafif, Florentia Kaguelidou, Sabina Kali, Smaragdi Kalomoiri, Darshana Hewa Kandamby, Chris Kandel, Ravi Kant, Dyah Kanyawati, Christiana Kartsonaki, Daisuke Kasugai, Anant Kataria, Kevin Katz, Simreen Kaur Johal, Christy Kay, Hannah Keane, Seán Keating, Andrea Kelly, Aoife Kelly, Claire Kelly, Niamh Kelly, Sadie Kelly, Yvelynne Kelly, Maeve Kelsey, Ryan Kennedy, Kalynn Kennon, Maeve Kernan, Younes Kerroumi, Sharma Keshav, Evelyne Kestelyn, Imrana Khalid, Antoine Khalil, Coralie Khan, Irfan Khan, Michelle E Kho, Saye Khoo, Yuri Kida, Peter Kiiza, Anders Benjamin Kildal, Jae Burm Kim, Antoine Kimmoun, Detlef Kindgen-Milles, Alexander King, Nobuya Kitamura, Paul Klenerman, Gry Kloumann Bekken, Stephen Knight, Robin Kobbe, Chamira Kodippily, Malte Kohns Vasconcelos, Volkan Korten, Caroline Kosgei, Arsène Kpangon, Karolina Krawczyk, Sudhir Krishnan, Oksana Kruglova, Deepali Kumar, Bharath Kumar Tirupakuzhi Vijayaraghavan, Pavan Kumar Vecham, Ethan Kurtzman, Neurinda Permata Kusumastuti, Demetrios Kutsogiannis, Galyna Kutsyna, Konstantinos Kyriakoulis, Marie Lachatre, Marie Lacoste, John G. Laffey, Marie Lagrange, Fabrice Laine, Olivier Lairez, Antonio Lalueza, Marc Lambert, François Lamontagne, Marie Langelot-Richard, Vincent Langlois, Eka Yudha Lantang, Marina Lanza, Cédric Laouénan, Samira Laribi, Delphine Lariviere, Stéphane Lasry, Odile Launay, Didier Laureillard, Yoan Lavie-Badie, Andrew Law, Cassie Lawrence, Teresa Lawrence, Minh Le, Clément Le Bihan, Cyril Le Bris, Georges Le Falher, Lucie Le Fevre, Quentin Le Hingrat, Marion Le Maréchal, Soizic Le Mestre, Gwenaël Le Moal, Vincent Le Moing, Hervé Le Nagard, Paul Le Turnier, Ema Leal, Marta Leal Santos, James Lee, Su Hwan Lee, Todd C. Lee, Gary Leeming, Bénédicte Lefebvre, Laurent Lefebvre, Benjamin Lefevre, Sylvie LeGac, Jean-Daniel Lelievre, François Lellouche, Adrien Lemaignen, Véronique Lemee, Anthony Lemeur, Gretchen Lemmink, Rafael León, Marc Leone, Michela Leone, François-Xavier Lescure, Olivier Lesens, Mathieu Lesouhaitier, Amy Lester-Grant, Bruno Levy, Yves Levy, Claire Levy-Marchal, Erwan L'Her, Gianluigi Li Bassi, Janet Liang, Geoffrey Liegeon, Wei Shen Lim, Chantre Lima, Bruno Lina, Andreas Lind, Guillaume Lingas, Sylvie Lion-Daolio, Keibun Liu, Marine Livrozet, Patricia Lizotte, Antonio Loforte, Navy Lolong, Diogo Lopes, Dalia Lopez-Colon, Anthony L. Loschner, Paul Loubet, Bouchra Loufti, Guillame Louis, Silvia Lourenco, Jean Christophe Lucet, Carlos Lumbreras Bermejo, Carlos M. Luna, Olguta Lungu, Liem Luong, Nestor Luque, Dominique Luton, Ruth Lyons, Olavi Maasikas, Oryane Mabiala, Samual MacDonald, Moïse Machado, Gabriel Macheda, Juan Macias Sanchez, Jai Madhok, Hashmi Madiha, Guillermo Maestro de la Calle, Rafael Mahieu, Sophie Mahy, Ana Raquel Maia, Lars S. Maier, Mylène Maillet, Thomas Maitre, Maximilian Malfertheiner, Nadia Malik, Paddy Mallon, Fernando Maltez, Denis Malvy, Victoria Manda, Jose M. Mandei, Laurent Mandelbrot, Frank Manetta, Julie Mankikian, Edmund Manning, Aldric Manuel, Ceila Maria Sant`Ana Malaque, Daniel Marino, Flávio Marino, Samuel Markowicz, Ana Marques, Catherine Marquis, Brian Marsh, Laura Marsh, Megan Marshal, John Marshall, Celina Turchi Martelli, Emily Martin, Guillaume Martin-Blondel, Alessandra Martinelli, Ignacio Martin-Loeches, Martin Martinot, Ana Martins, João Martins, Nuno Martins, Caroline Martins Rego, Gennaro Martucci, Olga Martynenko, Eva Miranda Marwali, Juan Fernado Masa Jimenez, David Maslove, David Maslove, Sabina Mason, Basri Mat Nor, Moshe Matan, Daniel Mathieu, Mathieu Mattei, Romans Matulevics, Laurence Maulin, Michael Maxwell, Javier Maynar, Thierry Mazzoni, Natalie Mc Evoy, Lisa Mc Sweeney, Lorraine McAndrew, Colin McArthur, Aine McCarthy, Anne McCarthy, Colin McCloskey, Rachael McConnochie, Sherry McDermott, Sarah E. McDonald, Aine McElroy, Samuel McElwee, Victoria McEneany, Allison McGeer, Chris McKay, Johnny McKeown, Kenneth A. McLean, Bairbre McNicholas, Elaine McPartlan, Edel Meaney, Cécile Mear-Passard, Maggie Mechlin, Omar Mehkri, Ferruccio Mele, Luis Melo, Joao Joao Mendes, Ogechukwu Menkiti, Kusum Menon, France Mentré, Alexander J. Mentzer, Emmanuelle Mercier, Noémie Mercier, Antoine Merckx, Mayka Mergeay-Fabre, Blake Mergler, Laura Merson, António Mesquita, Osama Metwally, Agnès Meybeck, Dan Meyer, Alison M. Meynert, Vanina Meysonnier, Amina Meziane, Mehdi Mezidi, Céline Michelanglei, Isabelle Michelet, Efstathia Mihelis, Vladislav Mihnovit, Hugo Miranda-Maldonado, Asma Moin, David Molina, Elena Molinos, Brenda Molloy, Mary Mone, Agostinho Monteiro, Claudia Montes, Giorgia Montrucchio, Sarah Moore, Shona C. Moore, Lina Morales Cely, Lucia Moro, Catherine Motherway, Ana Motos, Hugo Mouquet, Clara Mouton Perrot, Julien Moyet, Jimmy Mullaert, Fredrik Muller, Karl Erik Müller, Aisling Murphy, Aisling Murphy, Lorna Murphy, Marlène Murris, Srinivas Murthy, Himed Musaab, Dimitra Melia Myrodia, Dave Nagpal, Alex Nagrebetsky, Mangala Narasimhan, Nadège Neant, Holger Neb, Raul Neto, Emily Neumann, Bernardo Neves, Pauline Yeung Ng, Anthony Nghi, Duc Nguyen, Orna Ni Choileain, Niamh Ni Leathlobhair, Alistair Nichol, Prompak Nitayavardhana, Stephanie Nonas, Marion Noret, Lisa Norman, Alessandra Notari, Mahdad Noursadeghi, Karolina Nowicka, Saad Nseir, Jose I Nunez, Nurnaningsih Nurnaningsih, Elsa Nyamankolly, Fionnuala O Brien, Annmarie O Callaghan, Annmarie O'Callaghan, Giovanna Occhipinti, Derbrenn OConnor, Max O'Donnell, Tawnya Ogston, Takayuki Ogura, Tak-Hyuk Oh, Sophie O'Halloran, Katie O'Hearn, Shinichiro Ohshimo, Agnieszka Oldakowska, João Oliveira, Larissa Oliveira, Piero L. Olliaro, David S.Y. Ong, Wilna Oosthuyzen, Anne Opavsky, Peter Openshaw, Claudia Milena Orozco-Chamorro, Jamel Ortoleva, Javier Osatnik, Linda O'Shea, Miriam O'Sullivan, Nadia Ouamara, Rachida Ouissa, Clark Owyang, Eric Oziol, H M Upulee Pabasara, Maïder Pagadoy, Justine Pages, Amanda Palacios, Mario Palacios, Massimo Palmarini, Giovanna Panarello, Prasan Kumar Panda, Mauro Panigada, Nathalie Pansu, Aurélie Papadopoulos, Melissa Parker, Briseida Parra, Jérémie Pasquier, Bruno Pastene, Fabian Patauner, Luís Patrão, Patricia Patricio, Juliette Patrier, Lisa Patterson, Christelle Paul, Mical Paul, Jorge Paulos, William A. Paxton, Jean-François Payen, Miguel Pedrera Jiménez, Giles J. Peek, Florent Peelman, Nathan Peiffer-Smadja, Vincent Peigne, Mare Pejkovska, Paolo Pelosi, Ithan D. Peltan, Rui Pereira, Daniel Perez, Luis Periel, Thomas Perpoint, Antonio Pesenti, Vincent Pestre, Lenka Petrou, Ventzislava Petrov-Sanchez, Frank Olav Pettersen, Gilles Peytavin, Scott Pharand, Michael Piagnerelli, Walter Picard, Olivier Picone, Maria de Piero, Carola Pierobon, Carlos Pimentel, Raquel Pinto, Catarina Pires, Isabelle Pironneau, Lionel Piroth, Riinu Pius, Simone Piva, Laurent Plantier, Daniel Plotkin, Julien Poissy, Ryadh Pokeerbux, Maria Pokorska-Spiewak, Sergio Poli, Georgios Pollakis, Diane Ponscarme, Jolanta Popielska, Andra-Maris Post, Douwe F. Postma, Pedro Povoa, Diana Póvoas, Jeff Powis, Sofia Prapa, Sébastien Preau, Christian Prebensen, Jean-Charles Preiser, Anton Prinssen, Mark G. Pritchard, Gamage Dona Dilanthi Priyadarshani, Lucia Proença, Sravya Pudota, Oriane Puéchal, Bambang Pujo Semedi, Gregory Purcell, Luisa Quesada, Vilmaris Quinones-Cardona, Víctor Quirós González, Else Quist-Paulsen, Mohammed Quraishi, Christian Rabaud, Aldo Rafael, Marie Rafiq, Fernando Rainieri, Nagarajan Ramakrishnan, Blandine Rammaert, Ritika Ranjan, Christophe Rapp, Aasiyah Rashan, Thalha Rashan, Menaldi Rasmin, Indrek Rätsep, Cornelius Rau, Ali Raza, Andre Real, Stanislas Rebaudet, Sarah Redl, Brenda Reeve, Liadain Reid, Liadain Reid, Dag Henrik Reikvam, Renato Reis, Jordi Rello, Jonathan Remppis, Martine Remy, Hongru Ren, Hanna Renk, Liliana Resende, Anne-Sophie Resseguier, Matthieu Revest, Oleksa Rewa, Luis Felipe Reyes, Tiago Reyes, Maria Ines Ribeiro, David Richardson, Denise Richardson, Laurent Richier, Jordi Riera, Ana L Rios, Asgar Rishu, Patrick Rispal, Karine Risso, Nicholas Rizer, Chiara Robba, André Roberto, Stephanie Roberts, David L. Robertson, Olivier Robineau, Ferran Roche-Campo, Paola Rodari, Simão Rodeia, Julia Rodriguez Abreu, Bernhard Roessler, Pierre-Marie Roger, Emmanuel Roilideseira, Amanda Rojek, Juliette Romaru, Roberto Roncon-Albuquerque Jr, Mélanie Roriz, Manuel Rosa-Calatrava, Michael Rose, Dorothea Rosenberger, Andrea Rossanese, Matteo Rossetti, Bénédicte Rossignol, Patrick Rossignol, Stella Rousset, Carine Roy, Benoît Roze, Desy Rusmawatiningtyas, Clark D. Russell, Maeve Ryan, Maria Ryan, Steffi Ryckaert, Aleksander Rygh Holten, Isabela Saba, Musharaf Sadat, Valla Sahraei, Nadia Saidani, Maximilien Saint-Gilles, Pranya Sakiyalak, Leonardo Salazar, Gabriele Sales, Stéphane Sallaberry, Charlotte Salmon Gandonniere, Hélène Salvator, Olivier Sanchez, Angel Sanchez-Miralles, Vanessa Sancho-Shimizu, Gyan Sandhu, Zulfiqar Sandhu, Pierre-François Sandrine, Oana Sandulescu, Marlene Santos, Shirley Sarfo-Mensah, Bruno Sarmento Banheiro, Iam Claire E. Sarmiento, Benjamine Sarton, Sree Satyapriya, Rumaisah Satyawati, Egle Saviciute, Parthena Savvidou, Justin Schaffer, Tjard Schermer, Arnaud Scherpereel, Marion Schneider, Stephan Schroll, Michael Schwameis, Janet T. Scott, James Scott-Brown, Nicholas Sedillot, Tamara Seitz, Caroline Semaille, Malcolm G. Semple, Eric Senneville, Claudia Sepulveda, Filipa Sequeira, Tânia Sequeira, Pablo Serrano Balazote, Ellen Shadowitz, Mohammad Shamsah, Shaikh Sharjeel, Pratima Sharma, Catherine A. Shaw, Victoria Shaw, Haixia Shi, Mohiuddin Shiekh, Takuya Shiga, Nobuaki Shime, Hiroaki Shimizu, Keiki Shimizu, Naoki Shimizu, Sally Shrapnel, Hoi Ping Shum, Nassima Si Mohammed, Jeanne Sibiude, Atif Siddiqui, Louise Sigfrid, Piret Sillaots, Catarina Silva, Maria Joao Silva, Rogério Silva, Wai Ching Sin, Budha Charan Singh, Punam Singh, Pompini Agustina Sitompul, Vegard Skogen, Sue Smith, Benjamin Smood, Coilin Smyth, Michelle Smyth, Michelle Smyth, Morgane Snacken, Dominic So, Monserrat Solis, Joshua Solomon, Tom Solomon, Emily Somers, Agnès Sommet, Myung Jin Song, Rima Song, Tae Song, Jack Song Chia, Michael Sonntagbauer, Alberto Sotto, Edouard Soum, Ana Chora Sousa, Marta Sousa, Maria Sousa Uva, Vicente Souza-Dantas, Alexandra Sperry, B. P. Sanka Ruwan Sri Darshana, Shiranee Sriskandan, Sarah Stabler, Thomas Staudinger, Stephanie-Susanne Stecher, Ymkje Stienstra, Birgitte Stiksrud, Eva Stolz, Amy Stone, Adrian Streinu-Cercel, Anca Streinu-Cercel, Samantha Strudwick, Ami Stuart, David Stuart, Gabriel Suen, Jacky Y. Suen, Prasanth Sukumar, Asfia Sultana, Charlotte Summers, Dubravka Supic, Magdalena Surovcová, Konstantinos Syrigos, Jaques Sztajnbok, Konstanty Szuldrzynski, Shirin Tabrizi, Fabio S. Taccone, Lysa Tagherset, Ewa Talarek, Sara Taleb, Jelmer Talsma, Maria Lawrensia Tampubolon, Le Van Tan, Hiroyuki Tanaka, Taku Tanaka, Hayato Taniguchi, Coralie Tardivon, Pierre Tattevin, M Azhari Taufik, Hassan Tawfik, Richard S. Tedder, João Teixeira, Sofia Tejada, Marie-Capucine Tellier, Vanessa Teotonio, François Téoulé, Pleun Terpstra, Olivier Terrier, Nicolas Terzi, Hubert Tessier-Grenier, Adrian Tey, Vincent Thibault, Simon-Djamel Thiberville, Benoît Thill, Shaun Thompson, David Thomson, Emma C. Thomson, Duong Bich Thuy, Ryan S. Thwaites, Paul Tierney, Vadim Tieroshyn, Jean-François Timsit, Noémie Tissot, Maria Toki, Timo Tolppa, Kristian Tonby, Antoni Torres, Margarida Torres, Hernando Torres-Zevallos, Michael Towers, Théo Treoux, Huynh Trung Trieu, Cécile Tromeur, Ioannis Trontzas, Tiffany Trouillon, Jeanne Truong, Christelle Tual, Sarah Tubiana, Helen Tuite, Jean-Marie Turmel, Lance C.W. Turtle, Pawel Twardowski, Makoto Uchiyama, PG Ishara Udayanga, Roman Ullrich, Alberto Uribe, Asad Usman, Cristinava Vajdovics, Luís Val-Flores, Ana Luiza Valle, Amélie Valran, Stijn Van de Velde, Marcel van den Berge, Machteld Van der Feltz, Nicky Van Der Vekens, Peter Van der Voort, Sylvie Van Der Werf, Laura van Gulik, Jarne Van Hattem, Steven van Lelyveld, Carolien van Netten, Gitte Van Twillert, Noémie Vanel, Henk Vanoverschelde, Pooja Varghese, Michael Varrone, Charline Vauchy, Helen Vaughan, Aurélie Veislinger, Sebastian Vencken, Sara Ventura, Annelies Verbon, José Ernesto Vidal, César Vieira, Joy Ann Villanueva, Judit Villar, Pierre-Marc Villeneuve, Andrea Villoldo, Nguyen Van Vinh Chau, Benoit Visseaux, Hannah Visser, Chiara Vitiello, Fanny Vuotto, Chih-Hsien Wang, Jia Wei, Katharina Weil, Sanne Wesselius, Murray Wham, Bryan Whelan, Nicole White, Paul Henri Wicky, Aurélie Wiedemann, Surya Otto Wijaya, Keith Wille, Virginie Williams, Evert-Jan Wils, Ng Wing Yiu, Calvin Wong, Ioannis Xynogalas, Sophie Yacoub, Masaki Yamazaki, Yazdan Yazdanpanah, Cécile Yelnik, Stephanie Yerkovich, Toshiki Yokoyama, Hodane Yonis, Obada Yousif, Saptadi Yuliarto, Akram Zaaqoq, Marion Zabbe, Kai Zacharowski, Maram Zahran, Maria Zambon, Miguel Zambrano, Alberto Zanella, Konrad Zawadka, Hiba Zayyad, Alexander Zoufaly, David Zucman.
